# Hemiplegic Migraine in Children and Adolescents

**DOI:** 10.3390/jcm12113783

**Published:** 2023-05-31

**Authors:** Ilaria Bonemazzi, Francesco Brunello, Jacopo Norberto Pin, Mattia Pecoraro, Stefano Sartori, Margherita Nosadini, Irene Toldo

**Affiliations:** Juvenile Headache Center, Department of Woman’s and Child’s Health, University Hospital of Padua, 35128 Padua, Italy; ilaria.bonemazzi@aopd.veneto.it (I.B.); francesco.brunello@aopd.veneto.it (F.B.); jacoponorberto.pin@aopd.veneto.it (J.N.P.); mattia.pecoraro@aopd.veneto.it (M.P.); stefano.sartori@unipd.it (S.S.); margherita.nosadini@aopd.veneto.it (M.N.)

**Keywords:** hemiplegic migraine, familial hemiplegic migraine, sporadic hemiplegic migraine, HM, FHM, SHM, children, adolescents

## Abstract

Background: Only a few studies have focused on hemiplegic migraine (HM) in children despite its early age of onset. The aim of this review is to describe the peculiar characteristics of pediatric HM. Methods: This is a narrative review based on 14 studies on pediatric HM selected from 262 papers. Results: Different from HM in adults, pediatric HM affects both genders equally. Early transient neurological symptoms (prolonged aphasia during a febrile episode, isolated seizures, transient hemiparesis, and prolonged clumsiness after minor head trauma) can precede HM long before its onset. The prevalence of non-motor auras among children is lower than it is in adults. Pediatric sporadic HM patients have longer and more severe attacks compared to familial cases, especially during the initial years after disease onset, while familial HM cases tend to have the disease for longer. During follow-up, the frequency, intensity, and duration of HM attacks often decrease. The outcome is favorable in most patients; however, neurological conditions and comorbidities can be associated. Conclusion: Further studies are needed to better define the clinical phenotype and the natural history of pediatric HM and to refine genotype–phenotype correlations in order to improve the knowledge on HM physiopathology, diagnosis, and outcome.

## 1. Introduction

Hemiplegic migraine (HM) is a rare form of migraine with an aura characterized by transient motor weakness or hemiparesis (motor aura) [1]. HM is also associated with other non-motor aura manifestations (visual, sensory, aphasic, or basilar-type/brainstem symptoms) and with other symptoms typically accompanying migraine, such as nausea, vomiting, photophobia, or phonophobia [1]. A motor aura represents the peculiarity of HM compared to other forms of migraine with aura (MA), and its diagnostic criteria have been updated in the latest classification of headache disorders [1]. HM can be sporadic (SHM) or familial (FHM), with autosomal dominant inheritance [1].

SHM and FHM are similar with respect to epidemiology, trigger factors, clinical features, and neuroradiological and neurophysiological findings, but the two forms differ in terms of age of onset, genetics, and associated neurological picture [2,3,4].

Although a strong familial history is common among all types of migraine, HM is the only one in which monogenic conditions have been identified, and mutations in *CACNA1A*, *ATP1A2,* and *SCN1A* determine a significant number of familial clusters and sporadic cases. Nevertheless, other genes have been recently associated with HM, and in a relevant number of sporadic cases, it is possible that an underlying genetic cause is yet to be identified [2,5,6].

HM can affect both children and adults, with onset frequently occurring at a pediatric age [4,5,6,7,8,9,10,11]. Early transient neurological symptoms (isolated seizures, transient hemiparesis without a headache, and prolonged clumsiness after minor head trauma) can occur between the ages of 1 and 4 and long before the very first episode of HM is observed [5].

HM presents with similar clinical features both in adults and children; however, some remarkable differences have been reported. The prevalence of non-motor auras among children is lower than it is among adults [4,5]. In addition, the frequency, intensity, and duration of HM attacks often decrease during adulthood [2,7,12]. Moreover, HM prevalence in adults is greater among females than it is among males (6:1) [4], while gender representation is similar in children [5,13].

The aim of this review is to describe the peculiar features of pediatric-onset HM.

## 2. Materials and Methods

The search was conducted using PubMed and Mendeley by two independent research assessments (IB and FB), with the following queries: “hemiplegic migraine”, “HM”, “SHM”, and “FHM”. Only works in English language published from 2000 to April 2023 including children and adolescents (age range 0–18 years) with HM were included.

A total of 262 full-text articles were assessed for eligibility. Case reports and case series with fewer than 10 pediatric patients (and fewer than 2 families for the familial form) (*n* = 160) were excluded. Sixty-one articles reporting data different from those of interest were excluded. In the remaining 41 studies, narrative reviews (*n* = 7) and studies with incomplete data (*n* = 19) were excluded.

Finally, 15 studies based on both exclusively pediatric (*n* = 3) and mixed (children and young adults, *n* = 12) series were included.

The following data were collected: demographics, clinical features, neuroradiological, neurophysiological, and treatment data. To collect prevalence data, some of these works were based on questionnaires [4,12,13], semi-structured telephone interviews [2,3,14], or clinical interviews about the headache histories of patients [5,6,7,8,9,15].

We then conducted a narrative synthesis of the included studies as the available data did not allow us to perform a meaningful meta-analysis.

## 3. Clinical Diagnosis

HM is a subtype of migraine with aura, and the aura is characterized by motor weakness (or plegic symptoms), typically with a duration of less than 72 h [1]. The diagnostic criteria of the International Classification of Headache Disorders (ICHD-3) [1] are reported in Table 1.

Thomsen et al. [2,6] proposed some minor revisions to the diagnostic criteria, which are summarized in Table 2.

Basilar migraine (BM) or “migraine with brainstem aura”, is a type of MA clearly originating from the brainstem, but without motor weakness. BM may have at least two of the following fully reversible brainstem symptoms: dysarthria, vertigo, tinnitus, hypoacusis, diplopia, ataxia not attributable to the sensory deficit, and decreased level of consciousness (GCS < 13), but no motor or retinal symptoms [1].

Moreover, if at least one first- or second-degree relative has had attacks fulfilling the criteria for HM, a diagnosis of FHM can be conducted, otherwise the diagnosis of SHM is established [1].

## 4. Genetics and Relation with Other Migraine Types

Specific genetic subforms of FHM have been identified: FHM type 1 (FHM1) is associated with mutations of the *CACNA1A* gene (coding for a calcium channel) on chromosome 19 p13; FHM type 2 (FHM2) with mutations of the *ATP1A2* gene (coding for a K/Na-ATPase) on chromosome 1q23; and FHM type 3 (FHM3) with mutations of the *SCN1A* gene (coding for a sodium channel) on chromosome 2 [1]. The ICHD-3 has recognized each genetic subform (FHM1, 2, 3) as a distinct entity [1]. The diagnostic criteria are listed in Table 3.

ICHD-3 has also introduced the definition of “FHM, other loci”, in order to include all patients who fulfilled the clinical diagnostic criteria for FHM, but with no mutation detected on the *CACNA1A*, *ATP1A2*, or *SCN1A* genes using genetic testing [1].

Sporadic cases of HM can be determined using de novo mutations in FHM-related genes in patients without familial history of HM. The prevalence of *CACNA1A*, *ATP1A2*, and *SCN1A* mutations varies greatly among studies (7–63%) [4,5], mainly depending on the characteristics of the studied population. Pediatric cohorts and cohorts including only patients with early and severe disease onset showed a higher prevalence of gene mutations [5,13,16], while it was significantly lower in wider populations mostly including adult cases; for example, Hiekkala et al. found that 7% of the Finnish FHM families and none of the SHM patients had mutations in *CACNA1A* or *ATP1A2* genes [4].

For this reason, some authors recommend genetic screening in patients with HM onset below 6 years of age and in those with accompanying neurologic symptoms, due to a higher risk of carrying mutations in *CACNA1A* or *ATP1A2* genes in these cases [8,14]. Moreover, early paroxysmal motor or non-motor manifestations in childhood can precede the first HM attack, especially in mutated patients. These paroxysmal manifestations should be further investigated, especially in cases with a family history of HM [5].

Mutated patients could manifest a more severe phenotype: more extensive ictal motor weakness, attacks more often associated with confusion, brain edema, brainstem manifestations, abnormal neurological examination with mental retardation, and progressive ataxia [15]. A more frequent occurrence of HM triggered by mild head trauma is also reported in genetically positive patients [15].

Other rarer genes have been associated with HM clinical spectrum, such as *PRRT2* [17,18], *SLC4A4* [19], and *SLC1A3* [20]. Ten pediatric patients with Glut1 deficiency syndrome due to *SLC1A2* mutations showed HM-like attacks; only three were tested for typical HM genes and one showed a coexisting mutation in the *CACNA1A* gene. Further studies are needed to better understand if *SLC1A2* mutations alone could cause HM-like acute manifestation or if other secondary mutations are needed to determine the HM phenotype [21].

Interestingly, genes associated with HM are pleiotropic and different mutations can result in mixed clinical or familial phenotypes, including different paroxysmal neurological disorders [20]. The phenotypic spectrum of the main HM genes has been summarized in Table 4.

Despite all the remarkable findings about the genetic background of HM in recent years, the genetic cause still remains unknown in a significant proportion of HM cases and in the pediatric population. Polygenic interaction and/or multifactorial conditions could account for a considerable part of non-monogenic HM cases, as in other more common types of migraine [20]. However, there may be other unknown loci associated with HM [1].

The correlation between HM and more common types of migraine (MA, migraine without aura—MWA, BA), from a pathophysiological and genetic perspective is still debated [2,3]. However, patients with FHM have 7 times increased risk of MA than the general population [3]. In addition, 70% of SHM and 94% of FHM patients have one or more first-degree relatives with attacks of common MA, MWA, or both [5,7,11,12,14].

SHM patients have a significantly higher risk of MA but not of MWA [10,25].

Compared with the general population, first-degree relatives of probands exclusively having SHM exhibited a 2 times higher risk of MA [3].

Interestingly, mutations in HM-related genes were also found in common types of MA, MWA, and BM [7].

Ducros et al. reported that 11% (13/117) of subjects with mutations in the *CACNA1A* gene had no attacks of HM, in particular: 15% (2/13) of cases had no symptoms, 38.5% (5/13) were affected by MA, 8% (1/13) by MWA, 8% (1/13) by recurrent headaches with loss of consciousness, and 23% (3/13) had single transient episodes of unknown clinical significance (dysarthria, unilateral paresthesia, and confusion with fever) [2,8]. These findings could support the hypothesis that even the monogenic forms of SHM and FHM could represent a small part of a broader phenotypic spectrum in continuity with more common types of migraine [7].

## 5. Epidemiology, Time of Occurrence, and Natural History

HM is a rare condition. The only epidemiological systematic survey, based on the Danish population, found a SHM prevalence of at least 0.002% and a FHM prevalence of at least 0.003% [3,14].

Considering one of the studies with larger samples, the prevalence of HM stands at approximately 4.5% (406/9087) in all migraineurs [4,14] and at 12% (406/3383) when only MA cases are considered [4].

In most studies, SHM prevalence is similar to that of FHM [1,4,7,12,14,26].

The typical age of onset ranges between 3 and 55 years, with a mean onset age of about 11 years [4,5,6,7,8,9,10]; however, in some studies, it is beyond 17 years [2,3,11,14] and before 9 years in others [13,27]. Compared to MA, HM has more frequently a lower age of onset [2,14]. The lowest reported age of onset is of about 6 months [26].

Early transient neurological symptoms (i.e., prolonged aphasia during fever, isolated seizures, transient hemiparesis, and prolonged clumsiness after minor head trauma) can occur between ages 1 and 4 and long before the very first episode of HM [5].

The mean frequency of episodes in a lifetime also varies between subjects (from 1 per day to less than 5 during the entire lifetime), with a mean frequency of about 3 attacks per year [8,10,13,15]. 90% of patients experience at least 10 episodes during their lifetime [2,10]. The mean age at the last assessment in the literature is about 50 years [3,10], and the frequency and intensity of attacks often decrease in adulthood [3,9,14].

In pediatric patients, the frequency of episodes tends to be lower in SHM than in FHM, especially during the initial years after disease onset [5], whereas in adults, there is no significant difference in the frequency of attacks between FHM and SHM (1–2 per year). Prolonged attacks (>72 h) can characterize HM onset but also occur during disease evolution [5] and are reported in detail in Section 7.3.

Many pediatric studies report no significant gender difference in HM prevalence, both in SHM and FHM [5,6,7,8,9,13], but studies with mixed (children and adults) and larger cohorts describe a higher prevalence in females than in males, with an F:M ratio ranging between 2.5:1 and 6:1 [2,4,14,26]. This trend could be explained by the greater presence of adults in the studies and higher samples collected compared to the pediatric series with a limited study population. Migraine is 3 times more prevalent in female than in male adults, whereas the prevalence is comparable in both genders during childhood [28]. This can be probably due to the sharp decrease in estrogen-precipitated migraine attacks at this age [29,30].

Another hypothesis explaining gender differences between children and adults is that the studies with more samples and higher prevalence in females than in males also exhibit a lower frequency of the monogenic HM form. It is therefore possible that in the polygenic forms of HM the role of hormones is preponderant, as occurs in the common forms of migraine [5].

We outline the epidemiological characteristics of the literature populations in Table 5 for FHM, Table 6 for SHM, and Table 7 for mixed populations of FHM and SHM patients.

## 6. Trigger Factors

Up to 46% of patients report at least one trigger factor at the instance of the first HM attack [5].

Several factors can act as triggers for HM. Minor head trauma is frequently reported as a trigger for HM, both for SHM and FHM in its 3 subforms [2,5,6,8,10,11,12,13,15]. Overall, in HM patients, a history of minor head trauma is described in up to 45% cases [5,6]. On the other hand, it is rarely reported in patients with MA [25]. Emotional stress is the second-for-frequency possible trigger (up to 43%) [5,8,12].

Other possible trigger factors include: intense physical activity, previous catheter angiography, fever, heat/sun exposure, and lack of sleep; these precipitating factors are also frequently reported in MA and MWA [5,12].

The occurrence rate and features of trigger factors preceding the first HM attack do not seem to significantly differ between SHM and FHM patients [5,10].

Trigger factors of HM in the literature population are summarized in Table 8, Table 9 and Table 10.

## 7. Clinical Features

HM attacks are generally similar in SHM and FHM, with significant inter-individual variability [1,2,3,20], possibly due both to genetic and environmental factors [8].

In some cases, premonitory symptoms are reported, such as insomnia, yawning, fatigue, or irritability [9].

HM is also characterized by the presence of non-motor aura, which consists of a combination of 4 (with a reported prevalence of up to 72%), 3 (up to 30%), or rarely 2 (up to 5%) types of aura [3,14]; the most frequent temporal succession reported is visual, sensory, motor, aphasic, and basilar aura [2,3].

In some studies, the prevalence of non-motor auras (visual, sensitive, and aphasic) in children is described as remarkably lower than in adults [5]. These differences could be due to a data collection bias, children’s inability to describe all the aura symptoms, and finally to a change in aura characteristics with age [5].

Pediatric HM can have a heterogeneous clinical onset in SHM patients [5]; moreover, SHM has typically longer and more severe attacks compared to FHM [5].

### 7.1. Aura Features

Aura features in the study population are summarized in Table 8, Table 9 and Table 10.

#### 7.1.1. Motor Aura

Motor aura is mandatory for HM [1]; however, HM patients can also have attacks of MA or MWA [2,7,8,12]. Indeed, among patients with HM, 9.5–30% also suffer from MWA, 9–39% also suffer from MA, and 15% from both [2,7,8]. In addition, 5% of HM patients also report tension-type headache (TTH) [9,12].

Motor aura is characterized by weakness or a plegic deficit. Motor symptoms are most frequently localized in hands, arms, feet, legs, tongue, face, and body [2,3,4,10].

The progression of weakness is always gradual, requiring at least 5 min, and the irradiation of the symptom is usually unilateral [2,3,8,14] configuring a hemiparesis in most cases; however, there are also frequent reports of side-shifting progression of the motor deficit [3,10]. Motor aura tends to involve the same side of the body during every subsequent attack [2]. A bilateral motor aura can also, but rarely, occur [10].

#### 7.1.2. Sensory Aura

Sensory aura is typically characterized by hypoesthesia, numbness, or paresthesia, which irradiate gradually, sometimes presenting a hemisensory irradiation; these symptoms can be present in up to 97% of HM attacks [2,3,4,5,6,8,9,10,12,14]. As in the case of motor aura, sensory aura frequently shows unilateral localization but can shift from one side of the body to the other and occasionally develops bilaterally [2,3,14].

Sensory aura is more often localized in hands, arms, face, tongue, legs, and body [2,3,6], and it frequently involves the same side of the hemiparesis [2,3].

#### 7.1.3. Visual Aura

The frequency of visual aura in HM attacks ranges approximately between 74% and 97% [2,3,4,5,8,9,10,12,14]. It usually has a gradual progression, and it is often unilateral [2,3,14].

The visual symptoms include, in most cases, scintillating scotoma, phosphenes, flickering lines, blurred vision, hemianopia, and zig-zag lines, often impairing central vision [2,3,4,8,10,14].

#### 7.1.4. Aphasic Aura

Aphasic aura is common in HM, ranging between 60% and 81% [2,3,4,5,6,8,9,10,12,14]. It is characterized, in up to 95% of cases, by impaired language production, in particular aphasia, dysphasia, difficulties articulating speech, and sometimes difficulty finding the right words [2,3,4,6,7,8,9,14]. Occasionally, impaired comprehension is also observed [2,3,14].

#### 7.1.5. Basilar or Brainstem Aura

Basilar-type aura is present in about 60–73% of HM attacks [2,3,6,14].

The reported symptoms usually include dysarthria, loss of balance, clumsiness of hands or falling of objects from hands, tongue stiffness or numbness, vertigo, and diplopia, but rarer manifestations include decreased hearing, dizziness, decreased level of consciousness, tinnitus, “drop” attacks, pharyngeal numbness, swallowing difficulties, ear pressure sensation, or ear ache [2,3,4,7,12,14].

There are no differences in basilar-type symptoms of SHM compared to FHM [3].

Basilar-type symptoms can originate from the brainstem but more often originate due to the simultaneous dysfunction of the two hemispheres [2].

#### 7.1.6. Duration and Progression of Aura

Reported total duration of aura in HM is between 10 min and 7 days, but it more frequently ranges from 60 to 120 min [2,3,8,9,10,11,15], with about 5–30 min for each type of aura [2,3,14]; the most frequent temporal succession is visual, sensory, motor, aphasic, and basilar aura [2,3,14].

Duration is about 30 min to 24 h (mean duration 5 h) for motor aura, from 1 h to 12 h (mean duration 4 h) for sensory aura, from 5 min to 12 h (mean duration 2 h) for visual aura, and from 1 h to 12 h for aphasic aura [2,3,4,5,6,11,14].

Pelzer et al. reported a longer duration of aura in HM patients with a genetic mutation than in those without [6].

Eriksen et al. reported that the duration of the visual, sensory, and aphasic aura is almost never more than 24 h, but the motor aura lasted more than 24 h in 2% of patients with FHM and 8% of patients with SHM [14].

The progression time and the duration of the aura do not significantly differ in FHM and SHM [4,14].

#### 7.1.7. Non-Motor Aura of HM vs. Aura of MA

Non-motor aura symptoms and their order are similar in HM and MA, except for basilar-type aura which can be present only in HM [2,3]. In HM, 2 or more non-motor aura symptoms are constant unlike in MA [2,3]. If more than one aura symptom occurs in patients with MA, they always occur in succession, one at a time, different than in HM, in which they can occur simultaneously [3,4].

The gradual progression and the duration of the aura are not significantly different in HM and MA, but in some studies, HM aura had a longer duration than MA aura [3,14]. The duration of each aura symptom is similar in HM but the visual and sensory are considerably longer in HM compared to MA; the aphasic aura has a similar or longer duration in HM than in MA according to different studies [3,14].

The sensory aura tends to be more extended in HM than in MA, involving the face, arm, foot, and leg [14].

The visual aura is usually unilateral and consists of flickering lights similarly in HM and MA. In HM, the visual aura starts peripherally and consists of a scotoma, only rarely associated with a preserved central vision, while in MA, it starts centrally and more frequently consists of zig-zag lines [3,14].

Finally, in HM, the aphasic aura is more frequently characterized by impaired language production different to that in MA, in which impaired comprehension is more frequently reported [3,14].

### 7.2. Headache Features

In most studies, a headache is always present during a HM attack, but in some cases, aura symptoms can present without a headache [2]. Headache characteristics do not differ between genders [2,3]. Headache is described more often as unilateral (50–80%) but can also be bilateral (40–50%) [2,3,4,9,12,14]. Barros et al. reported frequent side-shifting irradiation [10]. Usually, headache is pulsating but sometimes can be also pressing/tightening, throbbing, or stabbing [2,3,4,10,12,14]. The intensity is almost always moderate or severe [2,3,4,10,12,14] and is very frequently aggravated by exercise [3,6,10,13].

All these features are not significantly different between FHM and SHM [2,3,4,14].

The headache characteristics fulfill the diagnostic criteria for MWA in 73% of patients with FHM and in 76% of patients with SHM [14].

#### 7.2.1. Associated Symptoms

The most frequently associated symptoms, similar to MWA, are nausea (84–94% of attacks), vomiting (58–80%), photophobia (70–99%), and phonophobia (70–92%) [2,3,10,12,14]. Fever (8–58%), disorientation, or confusion (36–81%) are also reported in HM attacks [1,4,5,6,11,12,14].

Sensitivity to odors, nasal congestion, lacrimation, eyelid swelling or turning red, motion sickness, Raynaud syndrome, loss of consciousness, and seizures can sporadically occur during HM attacks [5,6,11,15]. Particularly in children, irritability or agitation and drowsiness are also reported [5].

Finally, coma, visual hallucinations, meningism, pleocytosis, alien limb, apraxia, hyperacusis, and torticollis can rarely occur [7,12]. Pelzer et al. reported more frequent pleocytosis in genetically positive patients [6].

#### 7.2.2. Progression and Duration

During a HM attack, headache usually starts after the visual aura (usually before 60 min), but in some cases develops together with, and, in rare cases, before its onset [2,3]. This progression pattern could be explained by the theory that an HM attack begins with an unilateral cortical depression and then spreads to the brainstem and hemisphere in the late phase of the attack [1].

Headache frequently lasts between 4 and 72 h, but the duration can range between 10 min and 5 days [2,3,5,8,12,14].

#### 7.2.3. Headache Associated with HM vs. MA

Concerning the intensity and frequency of headaches, and associated symptoms, HM patients reported more severe, more frequent headaches and often had more accompanying symptoms compared to MA [3,4]. In addition, headache is more frequently aggravated by routine physical activity in SHM and FHM compared to MA [3].

The pulsating quality of headache is similar between HM and NHM (non-hemiplegic migraine) [4].

### 7.3. Prolonged HM Attacks

Prolonged attacks that last at least 72 h are reported in up to 20% of patients [2,3,5,14].

Prolonged motor aura (reversible hemiplegia lasting up to 4 weeks) is reported in both SHM and FHM, but in some studies, it is reported to be more frequent in FHM1 and FHM2 patients [2,3,8,12,31].

Additionally, attacks with prolonged aphasia (longer than 6 h) have been described in pediatric HM [2,3,5,14,32].

## 8. Associated Neurological Conditions

The neurological examination, outside HM attacks, is usually normal [2,7,9,10].

Some studies reported neurological signs in up to 60% of patients [2,5,8], with most frequent neurological alterations including cerebellar ataxia, nystagmus, postural tremor, and clumsiness [2,5,8].

In approximately 50% of FHM1 families, chronic progressive cerebellar ataxia occurs independent of the migraine attacks [1], as rarely observed in SHM [5]. HM patients have been reported to be affected by psychological disorders, allergies, asthma, Raynaud’s phenomenon, diabetes mellitus, stroke, and epilepsy more frequently than MA and MWA patients [4,6,12,13]. Few studies also described cognitive and motor delay, praxis difficulties, low-sustained attention, and obesity in HM cases [6,12,13].

Concerning epilepsy, Riant et al. reported that seizures may precede the onset of HM attacks by several years [13]. HM patients could have seizures during attacks (more frequently), or outside of them [6].

Barros et al. described the occurrence of HM and epilepsy in patients with FHM3, and they conducted functional studies whose findings showed that *SCN1A* gene mutation induced a gain-of-function that provoked neuronal hyperexcitability, potentially explaining the comorbidity [10]. Nevertheless, the reason for the same pathophysiological mechanism eliciting a seizure in one instance and a migraine in another remains to be elucidated [10].

## 9. Neuroradiological and Neurophysiological Findings

### 9.1. Neuroradiological Findings

Brain imaging, such as magnetic resonance (MR) imaging or computed tomography (CT), in patients with headache and neurologic signs can help in the differential diagnosis of intracranial hemorrhage, ischemic stroke, tumors, or abscesses. Neurovascular studies, such as MR-angiography or CT-angiography, can instead identify vascular occlusions, such as carotid dissection or cerebral venous sinus thrombosis [33].

In the 14 papers analyzed, only 5 provide neuroimaging findings of HM patients.

In the majority of cases, outside attacks, brain MRI was normal [7,10].

Outside HM attacks, the most significant brain MR findings consisted of white matter signal abnormalities on T2 sequences (up to 23%) [9,12]. Moreover, cerebellar atrophy was found both in patients with ataxia [2,12] and in those with no clinical cerebellar signs; in these cases, a mutation of the *CACNA1A* gene was detected [5,11].

During HM attacks, cytotoxic cortical unilateral (or partial) edema is documented both in FHM [5,12] and SHM [6,32]. In an SHM series, brain edema on MRI was most frequently found in genetically positive than in genetically negative patients [6]. Another study reported cortical edema in patients either with or without *CACNA1A* gene mutation (14/43 vs. 1/18) [6].

Cobb-Pitstick et al. documented, using MR angiography, vasospasm in the middle cerebral artery branches contralateral to the side of the weakness in a third of cases (4/12) [34]. The vasospasm resolved in 75% of cases, and in 12.5% of cases, vasogenic edema was observed in the corresponding parenchymal regions [27].

### 9.2. SPECT or PET

In the 14 papers analyzed, there are no reported functional findings in Positron Emission Tomography (PET) or Single Photon Emission Computed Tomography (SPECT).

Despite that, several studies have demonstrated abnormalities in advanced perfusion-based or nuclear imaging studies. Hyperperfusion may be found on MR perfusion-weighted imaging or SPECT, and vasodilation may be identified in noninvasive or conventional angiography. Other reports have found hypoperfusion without infarction, in some cases accompanied by vasoconstriction. These differences may be related in part to the timing of the imaging studies, as migraine aura is initially associated with transient hypoperfusion followed by hyperperfusion [27,35,36,37,38,39,40,41,42,43].

In a case of SHM with a very prolonged attack, studied with a multimodal approach (brain MRI, transcranial Doppler, and SPECT with 99mTc-ECD) in the acute and subacute phases, progressive changes of signal alterations in the affected hemisphere, temporally unrelated to the clinical course, were documented [32]. In particular, MR findings argued for a neuronal loss without cerebral ischemia, ^99m^Tc-ECD SPET documented a reversible impairment of the neuronal function, which lasted longer than the clinical symptoms and MR abnormalities [32]. The radiological and functional findings allowed to formulate a pathophysiological hypothesis, in which ion channel dysfunction caused prolonged neuronal depolarization, which could cause the shift of water from the extra- to the intra-cellular compartment and then cellular swelling and neuronal loss (see Figure 5 of Ref. [32]).

### 9.3. Electroencephalography (EEG)

In the 14 papers analyzed, only 4 provide neurophysiological findings of HM patients.

Patients with HM often have EEG abnormalities during or after the attacks (up to 85%) [6], mainly consisting of asymmetrical [5], occipital [9] slow-wave activity (66%) [5], especially in patients with positive genetics [6]. The slow-wave activity is often hemispheric, contralateral to the side of hemiplegia [27].

In some cases, the EEG trace is normal [10].

## 10. Differential Diagnosis

The most common differential diagnoses of HM include cerebrovascular events (transient ischemic attacks (TIA) or stroke) and epilepsy [1,3]. The gradual progression of motor symptoms due to spreading depression is the key feature helping the clinician distinguishing an HM attack from a cerebrovascular event [44]. Aura-like symptoms with motor weakness or plegy, with abrupt onset, followed or not by a headache require diagnostic caution and should be promptly investigated [14].

Other reported conditions that mimic HM are all conditions potentially associated with focal neurological deficits (i.e., hypercapnia, hyponatremia, hypocalcemia, hepatic failure, renal failure, meningitis/encephalitis, carotid dissection, antiphospholipid syndrome, systemic lupus erythematosus, and ornithine transcarbamylase deficiency). It is also important to consider inherited disorders associated with migrainous headache and hemiparesis: cerebral autosomal dominant arteriopathy with subcortical infarcts and leukoencephalopathy, mitochondrial myopathy, encephalopathy, lactic acidosis, and stroke-like episodes, hereditary hemorrhagic telangiectasia, a form of hereditary amyloid angiopathy, familial cerebral cavernous malformation, and benign familial infantile convulsions [3].

## 11. Treatment

There are no specific or standardized pharmacological treatments for HM and the therapeutic approach is similar to MA. Data concerning the safety and efficacy of different drugs and data about personalization of pharmacological treatment in relation to clinical phenotype and/or genetic diagnosis in patients with HM are limited, especially for pediatric patients. All data available come from case reports and small series; there are no randomized controlled trials for therapy of HM patients.

### 11.1. Acute Treatment

In the 14 papers selected for this review, only one [9] describes the use of ibuprofen and acetaminophen in acute attacks; however, data about the frequency of administration, side effects, and efficacy are not available.

Among experts, the use of triptans as acute abortion therapy for HM is still debated, due to their vasoconstricting effect. Nevertheless, the safety of the use of triptans in adolescent and adults with MA and MWA has been extensively demonstrated in recent years [45]. To date, there are no specific pathophysiological aspects of HM that could raise the suspicion that triptans may be specifically contraindicated in HM. Moreover, a study on a large retrospective cohort has shown an overall good safety and effectiveness profile of triptans used during HM attacks, except for a single report of a prolonged HM attack triggered by triptan administration [46]. The persisting mistrust of triptans in treating HM attacks could depend on the similarity between HM attacks and TIA/ischemic stroke at the disease’s onset, in which acute vasoconstriction is contraindicated. The use of triptans should probably be reconsidered, at least in non-severe HM attacks, in late childhood and adolescence [20].

Dexamethasone and hypertonic solution have been used in two CACNA1A-mutated patients as acute anti-inflammatory and anti-edema treatments during severe HM attacks, causing prolonged sensory impairment, cerebral edema, and encephalopathy. In both cases, corticosteroid treatment led to a rapid resolution of symptoms [47,48].

Ying et al. described a severe HM attack in an FHM2 patient characterized by headache, confusion, complete right hemiparesis, epileptic partial seizures, conscious disturbance, and fever. Brain MRI and magnetic resonance spectroscopy revealed unilateral extensive cerebral cortex edema, decreased N-acetylaspartate for neuronal damage, and increased lactate acid for mitochondrial dysfunction. Based on supposed pathogenic mechanisms of neuronal toxicity in ATP1A2 encephalopathy, an early acute treatment with memantine (NMDA receptor antagonist), idebenone, dl-3-n-butylphthalide (for protection of mitochondria function), flunarizine (traditional antimigraine drug), and oxcarbazepine (for partial seizures) was administered. All symptoms improved and recovery was observed within a few days, and a follow-up MRI showed complete disappearance of cortical edema [49].

### 11.2. Preventive Treatment

In the 14 papers included in this study, only two provide information about preventive treatment in HM patients. In a cohort of 46 children with HM [5], 20% of patients were under preventive treatment at the last follow-up (either with flunarizine or topiramate). Basheer et al. [26] showed that flunarizine is effective in reducing HM attack frequency by at least 50%; interestingly, its efficacy as a prophylactic treatment seems higher in HM (85% of responders) than in more common forms of MA (51%). Flunarizine showed a good safety profile, with 21% of treated patients experiencing mild-moderate side effects (mainly mood disturbance, increased appetite, and tiredness), leading to discontinuation of therapy in 17% of patients. The authors did not specify the criteria adopted to start preventive treatment.

Experience with other drugs comes from smaller studies and case reports. Verapamil has been tried with controversial results both as abortive and preventive treatment; moreover, data mainly comes from outdated studies on adults [38,50,51].

Valproate and lamotrigine have been used as preventive treatments in a family of three ATP1A2-mutated patients (FHM2). Each patient showed a reduction of HM attack frequency with valproate monotherapy and cessation of HM attacks after adding lamotrigine [52]. In a cohort of 59 MA patients, 8 patients with motor aura symptoms treated with lamotrigine obtained a significant reduction of motor aura frequency and intensity [53].

Preventive treatment with acetazolamide has been successfully used in FHM patients, even though it is not a common prophylactic drug used in other types of migraine. The mechanism of action which determines its efficacy on HM attacks is unknown. The efficacy of acetazolamide in treating other channelopathies (e.g., episodic ataxia type 2) caused by mutations in the same genes implicated in HM pathogenesis encouraged its use as a preventive treatment for FHM patients. However, data regarding acetazolamide are very limited and come from outdated studies [54,55].

In a recent retrospective study cohort, 9/11 HM patients showed a decrease in frequency and severity of both aura and headache after receiving onabotulinumtoxinA. Patients were administered onabotulinumtoxinA injections in a 12-week cycle; 6/9 responders experienced a “wearing off effect” of onabotulinumtoxinA around week 9 or 10 of the 12-week cycle, with improvements observed after the next administration [56].

## 12. Prognosis

In most cases, the frequency and intensity of HM attacks decreased in adulthood [3,9,14]; however, there are exceptions.

As previously reported, mutated patients could manifest a severe phenotype: more extensive ictal motor weakness, attacks more often associated with confusion, brain edema, brainstem manifestations, abnormal neurological examination with intellectual disability, and progressive ataxia [15].

## 13. Limitations

This study has several potential limitations. Firstly, a literature search was conducted using only two major electronic databases, PubMed and Mendeley. Therefore, additional studies could have been missed out. However, it is noteworthy that they are two of the largest available databases of medical studies.

Moreover, the incompleteness of the available quantitative data did not allow a meta-analysis study to be conducted. In addition, narrative and also systematic reviews use a retrospective observational research design, and as such they are subject to systematic and random errors.

The major challenge in the present work was to summarize all the features (demographic, clinical, neuroradiological, genetic, and treatment data) of HM despite the data heterogeneity of the 14 studies that investigated HM in the pediatric population.

Most of the studies considered a mixed series of children and adults, and in some instances, observations could be altered by the presence of a high number of adult cases; however, we tried to highlight the differences between pure pediatric studies and mixed ones.

Finally, we did not consider 3 studies in Japanese and Chinese languages, and we could have missed some pertinent sources.

Some of these works collected prevalence data relying on questionnaires; therefore, some values could be altered by the subjectivity of an autofill collection and not by means of a systematic approach.

## 14. Conclusions

In conclusion, HM is a rare, probably underdiagnosed, and underestimated condition that can occur in children even at a very early age. The first HM attack occurs long after transient and subtle neurological signs and symptoms are observed in early childhood, posing a challenge for child neurologists.

Pediatric HM is characterized by features and gender distribution that differ from adult HM, and the ICHD should take into consideration these issues in order to tailor the diagnostic criteria in childhood. A prompt differential diagnosis should be carefully undertaken in order to rule out other life-threatening acute neurologic conditions.

Pediatric HM has a heterogeneous clinical onset in SHM patients, despite showing longer and more severe attacks compared to FHM. Moreover, young patients with SHM tend to have a lower frequency of episodes compared to FHM cases, especially during the initial years after onset. In adults, this difference is not significant. Children present fewer non-motor auras as compared to adults.

Despite HM being characterized by severe attacks, it has a good prognosis in the majority of cases, but sometimes neurological conditions and comorbidities can be associated; therefore, an adequate follow-up is always necessary.

Further studies in the pediatric population are needed to better define the clinical phenotype and the natural history of HM and refine the genotype–phenotype correlations, in order to improve the knowledge of HM physiopathology, diagnosis, and prognosis.

## Figures and Tables

**Table 1:** ICHD-3 diagnostic criteria of HM [1].

HM Diagnostic Criteria
A. Attacks fulfilling criteria for ^1^ MA and criterion B below. B. Aura consisting of both of the following: 1. Fully reversible motor weakness; 2. Fully reversible visual, sensory, and/or speech/language symptoms.

^1^ MA = migraine with aura.

**Table 2:** Revisions of diagnostic criteria of HM proposed by Thomsen et al. [2,6].

HM Diagnostic Criteria Minor Revisions
Aura (B2 in Table 1) could consist of at least 2 symptoms instead of 3 A gradual development of aura (in at least 60 min) Presence of headache is not mandatory ^1^ BM could be included as a symptom

^1^ BM = basilar migraine.

**Table 3:** ICHD-3 diagnostic criteria of FHM1, FHM2, and FHM3 [1].

FHM 1	FHM 2	FHM 3
A. Attacks fulfilling criteria for FHM	A. Attacks fulfilling criteria for FHM	A. Attacks fulfilling criteria for FHM
B. A mutation on the CACNA1A gene has been demonstrated.	B. A mutation on the ATP1A2 gene has been demonstrated.	B. A mutation on the SCN1A gene has been demonstrated.

**Table 4:** Phenotypic spectrum of the main HM genes.

Gene	Phenotypic Spectrum
CACNA1A	^1^ HM, transient focal neurologic deficit without headache, coma after minor brain injury, progressive cerebella ataxia [10,22]
ATP1A2	HM, epilepsy, intellectual disability, prolonged HM attacks, confusion, and coma, ^2^ BM [12,23]
SCN1A	HM, epilepsy [24]
PRRT2	benign familial childhood epilepsy, episodic kinesigenic dyskinesia, familial childhood seizures with paroxysmal choreoathetosis [17]
SLC4A4	HM, renal tubular acidosis, glaucoma [19]
SLC1A3	HM [20]
SLC1A2	epilepsy—Glut1 deficiency syndrome, HM-like attacks [21]

^1^ HM = hemiplegic migraine. ^2^ BM = basilar migraine.

**Table 5:** Demographic features of the literature populations with FHM (of the 4 papers including pediatric cases).

Study	Number of Patients-Families	Adults (A)/ Children (C)	Mean Age of Onset/Range (Years)	Sex (%)	Altered Genes (%)
Ducros et al., 2001 [8]	104-19	A + C	11/1–51	50 F–50 M	CACNA1A: 100
Thomsen et al., 2002 [2]	147-44	A + C	17/1–45	71.4 ^1^ F–28.6 ^2^ M	CACNA1A: 7 ATP1A2: 7
Marconi et al., 2003 [9]	29-2	A + C	10.5/3–18	48.2 F–51.8 M	^3^ Different genes in FHM2 locus
Barros et al., 2014 [10]	10-2	A + C	11/6–18	40 F–60 M	SCN1A: 100

^1^ F = female. ^2^ M = male. ^3^ TAGLN2, APCS, CRP, IGSF8, ATP1A2, ATP1A4, OR10J1, GIRK3, KIR1.2.

**Table 6:** Demographic features of the literature populations with SHM (of the 4 papers including pediatric cases).

Study	Number of Patients	Adults (A)/ Children (C)	Mean Age of Onset/Range (Years)	Sex (%)	Altered Genes (%)
Terwindt, 2002 [11]	27	A + C	16/4–29	^1^ ns	CACNA1A: 7
Thomsen et al., 2003 [3]	105	A + C	21 ^2^ F, 16 ^3^ M /1–44	81.1 F-18.9 M	CACNA1A: 4 ATP1A2: 4
De Vries et al., 2007 [15]	39 (19 C, 20 A)	A + C	19/4–42	^1^ ns	CACNA1A: 3 ATP1A2: 13 SCNA1A: 3
Riant et al., 2010 [13]	25	C	7.7/1–15	56.5 F-43.7 M	CACNA1A: 32 ATP1A2: 44

^1^ ns = not specified. ^2^ F = female. ^3^ M = male.

**Table 7:** Demographic features of the literature populations with mixed FHM and SHM (of the 7 papers including pediatric cases).

Study	Number of Patients	Adults (A)/ Children (C)	Mean Age of Onset/Range (Years)	Sex (%)	Altered Genes (%)
Jurkat-Rott et al., 2004 [12]	FHM: 26 SHM: 24 ^1^ ns: 20	A + C	-/7 Month–41	ns	ATP1A2: 100
Kirchmann Eriksen et al., 2006 [14]	FHM: 147 SHM: 105	A + C	FHM: 17/1–45 SHM: 21/4–55	FHM: 71 ^2^ F-29 ^3^ M SHM: 81 F-19 M	ns
Cuenca-Leòn et al., 2008 [7]	FHM: 7 SHM: 7	A + C	10.9/8–18	57.2 F-42.8 M	CACNA1A and other variants
Basheer et al., 2012 [26]	FHM: 7 SHM: 8	C	13/1.5–17	ns	ns
Hiekkala et al., 2018 [4]	FHM: 131 SHM: 275	A + C	12/-	85.7 F-14.3 M	CACNA1A: 1 ATP1A2: 3
Pelzer et al., 2018 [6]	281 (212 ^4^ G+, 69 ^5^ G−)	A + C	G+: 10 G−: 12	G+: 54.5 F-45.5 M G−: 69.7 F-30.3 M	CACNA1A: 51 ATP1A2: 36 SCNA1A: 13
Toldo et al., 2019 [5]	FHM: 14 SHM: 32	C	FHM: 10.2/4–15 SHM: 10.6/2–16	FHM: 57 F-43 M SHM: 50 F-50 M	CACNA1A: 0 FHM, 33 SHM ATP1A2: 66 FHM, 57 SHM

^1^ ns = not specified. ^2^ F = female. ^3^ M = male. ^4^ G+ = patients with mutations in CACNA1A, ATP1A2, SCN1A. ^5^ G− = patients without mutations.

**Table 8:** Trigger factors of HM attacks and clinical features of aura in the FHM population.

Study	Trigger Factors (%)	Duration of Aura (%)	Motor Aura (M) (%)	Sensory Aura (S) (%)	Visual Aura (V) (%)	Aphasic Aura (A) (%)	Brainstem Aura (B) (%)
Ducros et al., 2001 [8]	Emotional stress (most frequent), minor head trauma (second most frequent)	a mean of 60 ^1^ m	^2^ F: 100 ^3^ L: Unilateral: 65	F: 93 Paresthesias and numbness	F: 74 Scintillating scotoma, phosphenes, hemianopia, blurred vision	F: 83 Various degrees of dysphasia	Nystagmus: 45, Ataxia: 34, Dysarthria: 9
Thomsen et al., 2002 [2]	Minor head trauma	1–4 m: *V*: 1.5 5–60 m: *M*: 42, *S*: 47, *V*: 72, *A*: 41.5 61–120 m: *M*: 25, *S*: 26, *V*: 16, *A*: 14 121–720 m: *M*: 19, *S*: 19, *V*: 9, *A*: 27 721–1440 m: *M*: 12, *S*: 7, *V*: 1.5, *A*: 7.5 >1440 m: *M*: 2, S: 1	F: 100 L: Unilateral: 100, ^4^ H: 59 vs. ^5^ NH: 41 Hand: 98, arm: 93, foot: 59, leg: 59, tongue: 57, face: 51, body: 31	F: 98 L: Unilateral: 100 Hand: 99, arm: 95, foot: 68, leg: 67, tongue: 83, face: 85, body: 35	F: 89 L: Unilateral: 100 Preserved central vision: 79, zig-zag lines: 3, flickering line: 50	F: 72 Problems articulating speech: 92, finding the right words: 66, understanding: 10, production: 96	F: 69 Diplopia: 51, decreased hearing: 48, dysarthria: 73, loss of balance: 72, vertigo: 72, decrease lever consciousness: 31, tinnitus: 29, drop attacks: 29
Marconi et al., 2003 [9]	Unknown	10 m–72 ^6^ h	F: 100	F: 93	F: 96.6	F: 82.8	^7^ ns
Barros et al., 2014 [10]	Stress: 20, pregnancy: 20, minor head trauma: 10	5–60 m: 90 61–540: 10	F: 100	F: 90	F: 80	F: 60	ns

^1^ m = minutes. ^2^ F = frequency. ^3^ L = localization. ^4^ H = hemiparetic. ^5^ NH = non-hemiparetic. ^6^ h = hours. ^7^ ns = not specified.

**Table 9:** Trigger factors of HM attacks and clinical features of aura in the SHM population.

Study	Trigger Factors (%)	Duration (%)	Motor Aura (M) (%)	Sensory Aura (S) (%)	Visual Aura (V) (%)	Aphasic Aura (A) (%)	Basilar-Type Aura (B) (%)
Terwindt et al., 2002 [11]	Minor head trauma: 7.4	5–60 ^1^ m: *M*: 56 up to ^2^ h: *M*: 20 up to 1 ^3^ d: *M*: 4 up to ^4^ ds: *M*: 12 up to 1 ^5^ w: *M*: 8	^6^ F: 100	^7^ ns	ns	ns	F: 8.7
Thomsen et al., 2003 [3]	ns	1–4 m: *V*: 1, *S*: 1, A: 1 5–60 m: *M*: 51, *S*: 51, *V*: 73, *A*: 68 61–120 m: *M*: 19, *S*: 23, *V*: 14, *A*: 11 121–720 m: *M*: 12, *S*: 14, *V*: 8, *A*: 12 721–1440 m: *M*: 10, *S*: 7, *V*: 4, *A*: 7 >1440 m: *M*: 8, S: 4, *A*: 1	F: 100 ^8^ L: Unilateral: 100, ^9^ H: 50 vs. ^10^ NH: 50 Hand: 99, arm: 92, foot: 50, leg: 50, tongue: 48, face: 45, body: 15	F: 98 L: Unilateral: 99 Hand: 100, arm: 95, foot: 59, leg: 60, tongue: 82, face: 93, body: 28	F: 91 L: Unilateral: 60 Preserved central vision: 2, zig-zag lines: 50, flickering line: 84	F: 81 Problems articulating speech: 89, finding the right words: 52, understanding: 5, production: 94	F: 81 Diplopia: 28, decreased hearing: 20, dysarthria: 69, loss of balance: 72, vertigo: 54, decrease lever consciousness: 19, tinnitus: 17, drop attacks: 12
De Vries et al., 2007 [15]	Minor head trauma: 5	5–60 m: *M*: 44 up to h: *M*: 33 up to 1 d: *M*: 3 up to ds: *M*: 14 up to 1 w: *M*: 6	F: 100	ns	ns	ns	F: 10.8
Riant et al., 2010 [13]	Minor head trauma: 24, stress: 8, fatigue: 4	10 m–ws	F: 100	ns	ns	ns	F: 28 nystagmus

^1^ m = minutes. ^2^ h = hours. ^3^ d = day. ^4^ ds = days. ^5^ ws = week. ^6^ F = frequency. ^7^ ns = not specified. ^8^ L = localization. ^9^ H = hemiparetic. ^10^ NH = non-hemiparetic.

**Table 10:** Trigger factors of HM attacks and clinical features of aura in the mixed FHM and SHM population.

Study	Trigger Factors (%)	Duration (%)	Motor Aura (M) (%)	Sensory Aura (S) (%)	Visual Aura (V) (%)	Aphasic Aura (A) (%)	Basilar-Type Aura (B) (%)
Jurkat-Rott et al., 2004 [12]	Minor head trauma, stress, physical exercise, heat/sun, angiography, lack of sleep	20–340 ^1^ m	^2^ F: 100	F: 84.6	F: 73.3	F: 62.5	F: 62.5 Dysarthria 40, decreased hearing: 20, vertigo: 10
Kirchmann Eriksen et al., 2006 [14]	^3^ ns	<5 m: *S*: 0^4^ f, 1^5^ s; *V*: 2f, 1s; *A*: 33f, 13s 5–30 m: *M*: 31f, 35s; *S:* 31f, 33s; *V*:60f, 41s; *A*: 43f, 45s 31–60 m: *M:* 30f, 19s; *S:* 36f, 20s; *V*: 34f, 28s; *A*: 18f, 19s >60 m: *M*: 86f, 51s; *S*: 77f, 49s; *V*: 35f, 26s; *A*: 53f, 28s	F: 100f, 100s ^6^ L: ^7^ U: 100f, 100s ^8^ H: 59f, 50s vs. ^9^ NH: 41f, 50s Hand: 98f, 99s; arm: 93f, 92s; foot: 59f, 50s; leg: 59f, 50s; tongue: 57f, 48s, face: 51f, 45s; body: 31f, 15s	F: 98 L: U: 100f, 99s Hand: 99f, 100s; arm: 95f, 95s; foot: 68f, 59s; leg: 67f, 60s; tongue: 83f, 82s; face: 85f, 93s, body: 35f, 28s	F: 91 L: Unilateral: 60 Preserved central vision: 79f, 2s, zig-zag lines: 3f, 50s, flickering line: 50f, 84s	F: 81 Problems articulating speech: 92f, 89s; finding the right words: 66f, 52s; understanding: 10f, 5s; production: 96f, 94s	F: 81 Diplopia: 51f, 28s; decreased hearing: 48f, 20s; dysarthria: 73f, 69s; loss of balance: 72f, 72s; vertigo: 72f, 54s; decrease lever consciousness: 31f, 19s; tinnitus: 29f, 17s; drop attacks: 29f, 12s
Cuenca-Leòn et al., 2008 [7]	ns	ns	F: 100	ns	ns	ns	F: 21 Dysarthria: 7, vertigo: 14
Basheer et al., 2012 [26]	ns	ns	F: 100	ns	ns	ns	ns
Hiekkala et al., 2018 [4]	Minor head trauma: 23	30 m–^10^ ws <5 m: *V*: 9 5–60 m: *M*: 37.5; *V:* 65 >60 m: *M*: 62.5; *V:* 19	F: 100 L: U: 88 H: 99 Face: 75, upper limb: 96, lower limb: 64	F: 90	F: 68.2 Scotoma: 74, scintillating scotoma: 70, photopsia: 50, blurring vision: 51	F: 89	F: 45.5 Basilar vertigo: 32, tinnitus: 28, decreased hearing: 27, diplopia: 27, swallowing difficulties: 22, unconsciousness: 16
Pelzer et al., 2018 [6]	Minor head trauma: 47 ^11^ G+, 4 ^12^ G−	*M* mean duration: G+: 60–120 m G−: 60–75 min	F: 100 H: 62G+, 43G− Hand: 2G+, 3G−; arm: 5G+, 13G−; arm and face: 2G+, 10G−, arm and leg 19G+, 28G−	F: 99G+, 96G−	F: 88G+, 99G−	F: 91G+, 80G−	F: 64G+, 45G−
Toldo et al., 2019 [5]	Stress: 43s, 43f; head trauma: 21s, 29f; physical effort: 21s, 14f; fever: 0s, 7f	*M*: s: 4.8 ^13^ h ± 10.1 h (5 m–48 h) f: 27.7 m ± 22.7 m (5–90 m)	F: 100	F: 57s, 83f	F: 29s, 50f	F: 11s, 8f	F: 57s, 75f

^1^ m = minutes. ^2^ F = frequency. ^3^ ns = not specified. ^4^ f = in FHM patients. ^5^ s = in SHM patients. ^6^ L = localization. ^7^ U = unilateral. ^8^ H = hemiparetic. ^9^ NH = non-hemiparetic. ^10^ ws = weeks. ^11^ G+ = patients with mutations in CACNA1A, ATP1A2, and SCN1A. ^12^ G− = patients without mutations. ^13^ h = hours.

## Data Availability

The data of the current study (not included in this published article) are available from the corresponding author upon reasonable request.
